# Impact of AtNHX1, a vacuolar Na^+^/H^+ ^antiporter, upon gene expression during short- and long-term salt stress in *Arabidopsis thaliana*

**DOI:** 10.1186/1471-2229-7-18

**Published:** 2007-04-05

**Authors:** Jordan B Sottosanto, Yehoshua Saranga, Eduardo Blumwald

**Affiliations:** 1 Department of Plant Sciences, University of California, One Shields Ave, Davis, CA 95616, USA

## Abstract

**Background:**

AtNHX1, the most abundant vacuolar Na^+^/H^+ ^antiporter in *Arabidopsis thaliana*, mediates the transport of Na^+ ^and K^+ ^into the vacuole, influencing plant development and contributing to salt tolerance. In this report, microarray expression profiles of wild type plants, a T-DNA insertion knockout mutant of *AtNHX1 *(*nhx1*), and a 'rescued' line (NHX1::*nhx1*) were exposed to both short (12 h and 48 h) and long (one and two weeks) durations of a non-lethal salt stress to identify key gene transcripts associated with the salt response that are influenced by *AtNHX1*.

**Results:**

147 transcripts showed both salt responsiveness and a significant influence of AtNHX1. Fifty-seven of these genes showed an influence of the antiporter across all salt treatments, while the remaining genes were influenced as a result of a particular duration of salt stress. Most (69%) of the genes were up-regulated in the absence of AtNHX1, with the exception of transcripts encoding proteins involved with metabolic and energy processes that were mostly down-regulated.

**Conclusion:**

While part of the *AtNHX1*-influenced transcripts were unclassified, other transcripts with known or putative roles showed the importance of *AtNHX1 *to key cellular processes that were not necessarily limited to the salt stress response; namely calcium signaling, sulfur metabolism, cell structure and cell growth, as well as vesicular trafficking and protein processing. Only a small number of other salt-responsive membrane transporter transcripts appeared significantly influenced by *AtNHX1*.

## Background

The *AtNHX1 *gene encodes the most abundant vacuolar Na^+^/H^+ ^antiporter in *Arabidopsis thaliana*, and mediates the transport of both K^+ ^and Na^+ ^into the vacuole [1,2]. Constitutive over-expression of *AtNHX1 *and homologues from other plants have been shown to confer significant salt tolerance in a variety of plant species as a result of increased vacuolar sequestration of sodium ions ([3], and references therein). The importance of *AtNHX1 *to salt stress tolerance was further demonstrated when T-DNA insertional mutant *nhx1 *'knockout' plants lacking a functional antiporter were shown to be more salt sensitive than wild-type Arabidopsis [4]. Additionally, it was found that *nhx1 *mutants exhibit an altered phenotype under normal growth conditions, including smaller cells, smaller leaves, and other developmental irregularities, associated with altered K^+ ^homeostasis brought about by the lack of AtNHX1. These results suggested that *AtNHX1 *is associated with other cellular processes that are not necessarily related to salt tolerance. Subsequently, the AtNHX1 coding region driven by the CaMV 35S promoter was introduced into the *nhx1 *knockout line. These 'rescued' plants (NHX1::*nhx1*) displayed AtNHX1 activity, and a phenotype similar to that of wild-type plants [4].

The transcriptional profile of the *AtNHX1 *'knockout' (*nhx1*) line has been analyzed previously [5]. That study examined the differences in transcript level using the Affymetrix^® ^23 k 'Full Genome' GeneChips^® ^to look at the differences of expression levels between wild-type and *nhx1 *plants grown in the absence of salt stress, and also to examine the difference in relative gene expression changes that occurred after exposure to two weeks of salt stress. It was found that there was little overlap between the two comparisons suggesting that the role of the antiporter as part of the salt stress response machinery is distinct from its role under normal growing conditions. The previous study [5] also suggested that *AtNHX1 *is important to the expression of several cellular processes, including components of cell structure, protein processing and trafficking, and energy balance, although *AtNHX1 *did not appear to dramatically affect the expression of many other transporters.

This report further establishes and clarifies the influence of AtNHX1 on gene expression, limiting the analysis to only those transcripts that respond to salt stress, and including an analysis of the influence of both shorter (12 h and 48 h) and longer (one week and two weeks) salt stress treatments. Additionally we have employed an NHX1::*nhx1 *'rescued' line to determine transcripts whose expression levels correlate with the expression of *AtNHX1*. This approach provides evidence of the influence of a single gene on the expression of other genes while helping to eliminate some of the non-specific effects that result from the mutation of the antiporter.

## Results and discussion

Plants have been shown to have a "dual response" to salt stress, with an early response to the osmotic stress brought about by the more negative water potential of a salty soil solution, and a later response due to the Na^+ ^toxicity resulting from the relatively slower entry of Na^+ ^ions into the leaf tissues [6]. In an effort to include both components of the salt-stress response, we studied the influence of AtNHX1 on gene expression after 12 hours, 48 hours, one week, and two weeks of salt stress. This work is an extension of a previous microarray study that compared wild-type and nhx1 "knockout" plants before and after 2 weeks of salt stress [5]. Here the added shorter salt stress treatments (12 hours, 48 hours, and one week) and the inclusion of the NHX1::*nhx1 *'rescued' line allowed for a more detailed analysis of the importance of AtNHX1 to the expression of salt responsive genes. Furthermore, the greatly increased number of microarray chips used here (increased from 14 to 48) allowed for the use of a more robust ANOVA-based statistical analysis.

The NHX1::*nhx1 *plant line used in this study has an average increased expression of 50% of *AtNHX1 *as compared to the wild-type. This level of expression were sufficient to restore the wild-type phenotype [4], but was insufficient to confer meaningful salt tolerance [1]. Also, because *AtNHX1 *is normally expressed in all tissues and to a comparable level in all cells, with the exception of meristematic cells lacking vacuoles [4,7,8], expression patterns under a constitutive promoter should not differ dramatically from expression under the native promoter. The objective behind using this line was to identify transcripts with expression directly affected by the presence or absence of a functional AtNHX1.

### Overview of salt-responsive transcripts influenced by *AtNHX1*

Out of the 17,030 genes that exhibited reliable expression data, 4,027 transcripts met the criteria of salt responsiveness, and 147 of these also showed a significant influence by AtNHX1, as delineated in Materials and Methods. This study focused on transcripts that showed a significant influence by both salt and AtNHX1. Other transcripts also influenced by AtNHX1 but not responding to the salt treatments, or responding to salinity but without restored levels of expression in the NHX1::*nhx1 *were not considered. The latter transcripts may yet be an important component of *AtNHX1*-related processes, but due to inherent variation in expression levels or the consequences of constitutive AtNHX1 expression, they did not meet the necessary significance criteria threshold to establish a clear relationship to the presence of the antiporter. Even with an increased statistical filtering, comparisons of more salt treatments, and an analysis of salt responsive transcripts based on absolute values rather than relative values, 42 of the 147 (>28%) transcripts that showed a significant effect of *AtNHX1 *in this report, were also previously shown to have an influence of *AtNHX1 *on expression levels [5] (comparison data not shown).

Among the 147 salt-responsive transcripts that were significantly affected by AtNHX1, 102 genes (69%) were up regulated while only 44 genes (31%) were down regulated in the absence of AtNHX1, with one transcript (At3g54810) showing increased expression after one week of salt stress, but decreased expression after two weeks of salt stress. The Genevestigator^® ^database [9,10] was searched and most (88%) of same transcripts were found to have at least a 20% change in expression in response to salt, drought, and/or osmotic stress, despite differing stress and growing conditions.

**Figure 1:**
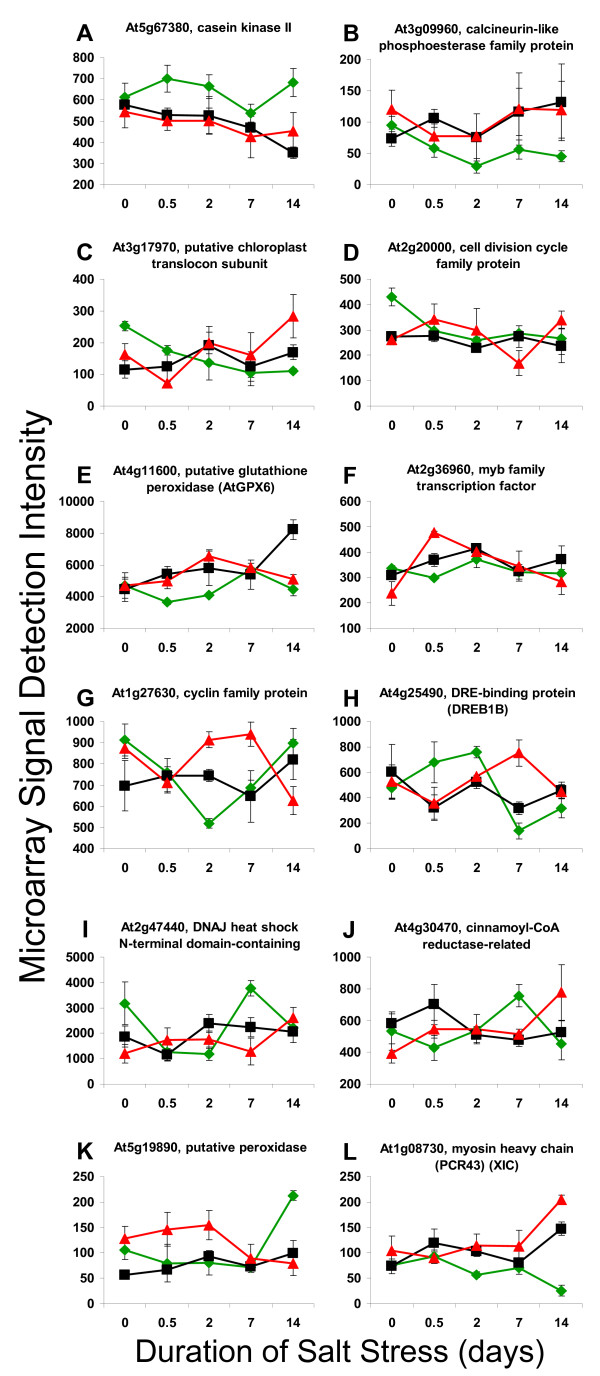
Expression profiles of selected salt responsive transcripts showing a significant influence of the AtNHX1 cation/H^+ ^vacuolar antiporter. Transcripts that were found to be influenced by *AtNHX1*: **[A,B] **regardless of specific salt treatment, or **[C,D] **specifically under control conditions; **[E,F] **12 h salt treatment; **[G,H] **48 h treatment; **[I,J] **one week treatment; **[K,L] **two weeks treatment. Green ◆ = *nhx1*, Black ■ = wild-type, Red ▲ = NHX1::*nhx1*. Values are the Mean ± S.D. (n = 4 for control, n = 3 for all other treatments).

Fifty-eight of these 147 genes showed an influence of the antiporter across all salt treatments (significant effect only of genotype; see examples in Figure 1A, B) with the other 89 transcripts showing differential expression due to the presence of AtNHX1 under a specific salinity treatment (genotype × treatment interaction). The latter 89 transcripts were influenced by *AtNHX1 *typically only in one treatment (three transcripts showed a specific influence of two treatments), with fewer transcripts showing this pattern under control conditions (12 transcripts; e.g. Fig. 1C, D) or after the shortest salt treatment of 12 hours (15 transcripts; e.g. Fig. 1E, F) as compared with longer exposure to salinity (20–24 transcripts per treatment; e.g Fig. 1G–L). The two-factor ANOVA used in this study to determine the influence of AtNHX1 is considered a powerful tool for the analysis of microarray experiments with multiple factors [11], as it utilized all 48 microarray data points to distinguish between an effect of genotypes across all treatments (main effect) and a treatment-dependent effect of lines (genotype × treatment interaction). In order to focus on AtNHX1-influenced salt-responsive genes, a further statistical test was used to identify transcripts with significantly different expression levels in the *nhx1 *line relative to both wild type and NHX1::*nhx1 *lines. While AtNHX1 influenced the expression of 58 genes that were not specific to a particular salt treatment, most salt-responsive genes appeared significantly impacted in conjunction with a particular length of salt stress, with more genes influenced as the duration of stress was increased. This pattern would suggest that AtNHX1 has greater impact on the expression of other genes as the influence of salt stress shifts from initial osmotic stress to the ion stress [6].

**Figure 2:**
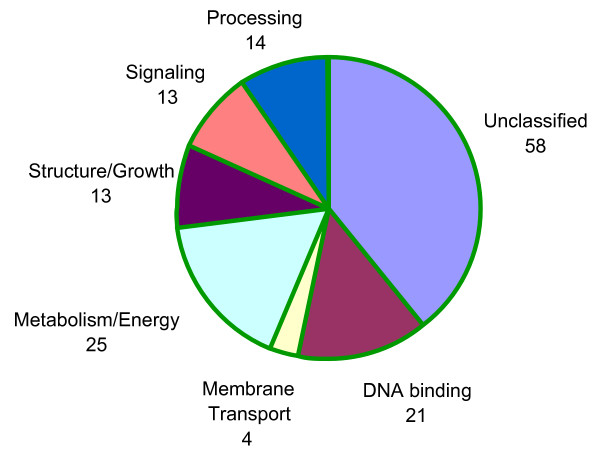
Functional assignments of transcripts influenced by AtNHX1. Pie chart depicting the functional distribution of all 147 transcripts showing a significant influence of the AtNHX1 cation/H^+ ^antiporter.

Various databases were queried [12-14] to determine the most likely functional role of the proteins encoded by the 147 salt-responsive transcripts showing an impact of AtNHX1 on their expression levels. These transcripts were then classified into general functional groups to assist with the analysis. (Figure 2) The largest group of transcripts showing the influence of the AtNHX1 vacuolar antiporter was comprised of 58 genes (40%) with unclear functional classifications (Additional file 1) Interestingly, the percentage of unclassified transcripts was larger among the up-regulated genes (46% of the total increased) than among the down-regulated (26% of the total decreased), suggesting that more novel salt-responsive genes are increasing in the absence of functional AtNHX1.

The remaining 89 transcripts encode proteins from a variety of functional groups. The majority of encoded proteins included signaling elements, DNA binding elements, components of the protein processing and trafficking machinery, and enzymes involved with metabolic and energy balance of the cell. Details of all salt-responsive transcripts that also showed a significant influence of AtNHX1 are presented in Table 1. Specific transcripts of particular interest are discussed in the subsequent sections of this report. The research community is encouraged to explore the data for all transcripts that were found to have meaningful expression levels [15].

### *AtNHX1 *influences salt-responsive transcripts encoding signaling elements, including several putative calcium-binding proteins

Thirteen salt-responsive signaling-associated transcripts were significantly influenced by the AtNHX1 antiporter (Table 2A). Nine of these transcripts exhibited significantly increased expression levels in the *nhx1 *line, while the expression of 4 transcripts showed reduced expression. Six of the up-regulated transcripts showed a genotype × treatment interaction with a significant effect of AtNHX1 being observed only after a week or more of salt treatment, suggesting that cellular signaling was not strongly impacted by AtNHX1 until the later stages of salt stress. The only transcripts that displayed a general trend of increased expression for all salt treatments were three kinases. These included two receptor protein kinases (At4g04540 and At5g56040) and a casein kinase II (At5g67380) all with unknown roles, although a CK2 homolog, with unidentified targets, has been implicated in the response of maize to ABA [16].

A notable feature of the signaling elements influenced by AtNHX1 is the number of transcripts encoding calcium-binding proteins, including 2 of the 9 transcripts that were up-regulated (At5g66210 and At1g52570) and 3 of the 4 transcripts (At3g09960; At2g38750; At4g34150) down-regulated in the *nhx1 *line. At5g66210 is a calcium-dependent protein kinase with an undetermined role, that is localized at the plasma membrane [17]. At1g52570 is a phospholipase D, shown to have regulatory functions in plant growth and development as well as the stress response (reviewed in [18]). The signaling transcripts with diminished expression in the *nhx1 *line included a member of the annexin family, *ANNEXIN4 *(At2g38750/AnnAt4). Annexins are Ca^2+^-dependent membrane-binding proteins found in most eukaryotic species, playing roles in a wide variety of cellular processes. In *Arabidopsis*, they have been implicated, though not necessarily limited to, roles in Golgi-mediated secretion [19] which is also one of their key roles in animal systems. Moreover, AnnAt4, along with AnnAt1, have been shown to be important in Ca^2+^-dependent signaling in response to osmotic stress and to ABA [20]. The other calcium-binding signaling components with diminished expression in the *nhx1 *line included At4g34150, a transcript encoding a protein that is similar to calcium-dependent protein kinases and contains a C2 domain (Ca^2+^-dependent membrane-targeting module often associated with signal transduction or membrane trafficking, [21]) and At3g09960, a calcineurin-like phosphoesterase family member [22].

The presence of several calcium binding elements provides further evidence of the influence of pH and ion homeostasis on the calcium signaling network. Calcium has been shown to be an important component of the SOS (Salt Overly Sensitive) network, with a calcium-binding protein (SOS3) in conjunction with a kinase (SOS2), influencing both the expression and activity of the SOS1/AtNHX7, a plasma membrane Na^+^/H^+ ^exchanger that is important to salt stress tolerance and cytosolic pH homeostasis [23] A previous microarray study has also shown that Ca^2+ ^starvation induced decreased expression of AtNHX1, AtNHX2 and AtNHX5 in *Arabidopsis *[24], further suggesting a link between vacuolar cation/H^+ ^antiporters and calcium levels in the cell. Moreover, the C-terminal portion of AtNHX1 itself has been shown to bind a calmodulin-like protein, with activity and ion specificity modified by the interaction, in a calcium- and pH-dependent manner [3]. Our results provide further demonstration of the influence of Ca^2+ ^on cellular ion and pH homeostasis.

### *AtNHX1 *influences the expression of DNA binding elements including water deficit responsive transcripts

The expression of 20 salt-responsive transcripts encoding DNA binding elements (mostly transcription factors) was influenced by AtNHX1 (Table 2B). Similar to the trends seen among the signaling elements discussed above, most (80%) of the transcription factors exhibited increased expression in *nhx1 *plants and the majority of the individual transcripts were influenced by a specific salt treatment. Genes encoding DNA binding elements were affected by AtNHX1 in response to both short and long terms of salt exposure whereas signaling elements were predominately influenced after longer treatments with salt. Several of these genes have been shown to be associated with the plant response to osmotic stress. At4g25490/CBF1 and At1g21910, which displayed increased expression in the *nhx1 *line are members of the DREB transcription factor family shown to be involved in the response of plants to different environmental stimuli by binding to dehydration-responsive element (DRE) promoter regions of stress-inducible genes [25]. CBF1, also known as DREB1B, has been shown to be involved in increasing tolerance to low temperatures, and shows a response to ABA treatment [26], and was also recently shown to be regulated by the circadian clock [27]. Conversely, expression of At4g27410/RD26 was reduced in the *nhx1 *plants. RD26 is a drought- and salt-induced transcript belonging to the NAC gene family, that is also part of an ABA-dependent stress-signaling pathway [28]. The altered expression of these transcripts highlights the impact of AtNHX1 on known and predicted components of drought stress-related pathways.

Another transcript with an established role in the environmental stress response and influenced by the presence of the AtNHX1 was a transcriptional co-activator, At3g24500/AtMBF1c, that exhibited a 3–4 fold increase in expression as a result of the *nhx1 *mutation with 12 hours of salt stress. Over-expression of AtMBF1c in *Arabidopsis *enhanced the tolerance of the plants to different stresses (including osmotic), possibly due to perturbation of the ethylene-response signal pathway [29]. Moreover, plants over-expressing AtMBF1c demonstrated increased expression of several genes (At5g66210, At1g21910, At1g35140, At4g08950, At1g28480, and At2g32150) [29] that were also shown to be significantly influenced by AtNHX1 in this study, suggesting a possible relationship between altered ion homeostasis and stress-induced hormonal responses.

A heat shock transcription family member (At2g26150/AtHsfA2) showed a significant influence of AtNHX1 after 12 hours of salt stress. The altered level of expression of this gene may reflect another aspect of the disrupted response to stress in the *nhx1 *line. However it is also possible that this gene is part of the protein processing network that is disrupted in the absence of AtNHX1 (see following discussion).

Other AtNHX1-influenced transcripts encoding putative DNA binding elements have not been associated with abiotic stress response previously. At3g56980/OBP3, which increased in expression after 48 hours of salt treatment, is a transcription factor shown to target genes that are inducible by salicylic acid, and is important to normal plant development [30]. At5g56860, a GATA-type zinc finger family member also influenced by AtNHX1 in a salt-independent manner, has been shown to be induced by nitrate, and to be important to chlorophyll synthesis and glucose sensitivity [31]. Another GATA-type zinc finger family member (At3g54810/BME-ZF) was also influenced by AtNHX1 significantly following at one week of salt stress. Although the role of this transcript in adult plants is not clear, BME-ZF has been shown to act as a regulator of seed germination during cold stratification [32], which may reflect a role in the response to environmental stimuli similar to other GATA-type genes.

The *nhx1 *plants have been shown to have altered leaf development, in addition to increased salt sensitivity [4], and the expression of several transcription factors associated with leaf morphology and development were influenced by AtNHX1. While most developmental genes are expected to be independent of salinity effect, two genes were significantly influenced by AtNHX1 under specific salt treatments. The expression of At2g36960, encoding the *TOUSLED *gene, was decreased in the *nhx1 *line after 12 hours of salt stress. TOUSLED interacts with chromatin regulators and its expression normally increases in dividing cells [33]. In addition, At4g00850/AtGIF, involved in leaf growth and morphology [34] showed a significant effect of AtNHX1 after two weeks of salt stress. Possibly, these factors contribute to the altered gene expression that is associated with the *nhx1 *phenotype [4].

### *AtNHX1 *is associated with sulfur metabolism

Of the 89 AtNHX1-influenced transcripts with an assigned or putative function, 25 transcripts, found on Table 2C, encode genes with metabolism or energy functions not directly associated with cell structure or cell growth (discussed in the next section). The majority of these transcripts had significantly lowered expression in the *nhx1 *line, in contrast to the overall patterns of genes showing mostly increased expression in the absence of AtNHX1. This pattern would suggest an overall decrease of metabolism- and energy processes-related genes in the knockout plants.

Twelve of the 18 metabolism/energy-related transcripts down-regulated in the *nhx1 *plants were generally decreased in the *nhx1 *line over all treatments. On the other hand, the transcripts with increased expression in *nhx1 *plants were responsive to particular lengths of salt stress. These results indicated that, though in general gene expression was enhanced in the *nhx1 *line to compensate for altered ion homeostasis, metabolic and energy processes were compromised in the absence of *AtNHX1*.

At least 5 of the 12 transcripts with diminished expression over all salt treatments in the *nhx1 *line appeared to be associated with sulfur/sulfate metabolism pathways. Transcripts encoding adenosine-5'-phosphosulfate-kinase (At4g39940/AKN2), a 5'-adenylylsulfate reductase/PAPS reductase homolog (At4g04610/APR1/PRH19), and a homocysteine methyltransferase (At3g22740/HMT3) have well established roles in sulfur metabolism [35]. The diminished expression of these transcripts would suggest a decrease in the synthesis of both glucosinolates and methionine within the leaves of the *nhx1 *plants. Other sulfur-related transcripts were also diminished over all treatments in the *nhx1 *line, encoding a glutathione S-transferase (At5g17220/AtGSTF12) a putative glutaredoxin (At1g28480), and CYP79F1 (At1g16410) a protein that mediates the formation of glucosinolates that are derived from methionine [36]. Additionally, a glutathione peroxidase (At4g11600/AtGPX6), which is known be regulated by abiotic stress [37], was down-regulated in the *nhx1 *line specifically with 12 hours of salinity stress.

There are several other down-regulated transcripts that are also likely to play a role in sulfur assimilation pathways. OPR3 (At2g06050) catalyzes the middle step in jasmonic acid biosynthesis, has been associated with the plant response to environmental stresses, and influence the sulfur metabolic pathway [38]. These results highlight a link between S-assimilation/metabolism and the expression levels of the AtNHX1 antiporter, as also suggested by a study using transgenic *Brassica *plants overexpressing *AtNHX1 *[39].

### *AtNHX1 *influences cell wall metabolism and components of cell growth

Thirteen salt-responsive, AtNHX1-influenced transcripts, were associated with cell wall metabolism and cell growth (Table 2D). Nine of these exhibited increased expression in the *nhx1 *plants, mostly after exposure to salt stress of two days or longer. The up-regulated cell wall-associated genes included At5g57560/TCH4 – encoding an endo-xyloglucan transferase that has been shown to be rapidly up-regulated in response to many environmental and hormonal stimuli [40], a galactosyltransferase (At3g62720), a galacturonosyltransferase (At3g02350), a polygalacturonase family member (At1g19170), a putative cinnamoyl-CoA reductase (At4g30470), and a 3-deoxy-D-manno-octulosonate 8-phosphate synthase (At1g16340). Transcripts encoding proteins with cell-wall associations also had diminished expression in the *nhx1 *line, including two cellulose synthase-like genes (At4g16590 and At1g24070) that were diminished with all treatments, and a pectinacetylesterase (At1g57590) transcript that was diminished after two weeks of salt stress.

The altered expression of the above-mentioned transcripts associated with cell size and structure, in addition to some of the transcription factors mentioned earlier, are likely to be involved in the altered developmental phenotype of the *nhx1 *line, showing smaller cells, smaller leaves and diminished growth [4]. There are also four salt responsive transcripts displaying altered expression levels in the absence of the AtNHX1 that are part of cell expansion and growth. Under control conditions a cell division gene (At2g20000/HBT) has increased expression in the *nhx1 *line whereas with 48 hours of salt stress a cyclin family protein (At1g27630) shows decreased expression. Two putative expansins also show increased *nhx1 *expression levels (At2g40610/AtExpA8 and At3g45970/AtExlA1) at 12 hours and one week of salt stress, respectively. Intracellular ion and pH homeostasis is important to the regulation of cell volume and cell cycle progression [41,42], and in mammalian systems, calcium-regulated sodium/proton exchange activity has been implicated in carcinogenesis and proliferation [43,44]. The diminished cell size of plants lacking AtNHX1 [5] can be a consequence of the roles played by AtNHX1 in ion and pH homeostasis, and the influence of the antiporter on calcium signaling and vesicular trafficking processes (discussed below). Whether the absence of functional AtNHX1 can change the rate of cell proliferation remains to be demonstrated.

### *AtNHX1 *influence the expression of protein processing and trafficking components in response to salt stress

Fourteen of the AtNHX1-influenced salt-responsive genes appeared to play roles in the processing and trafficking of other cellular components and proteins (Table 2E). Nhx1, the yeast orthologue of AtNHX1, has been shown to play an important role in protein trafficking in yeast [45,46], and the regulation of endosomal pH by Nhx1 controls the vesicle trafficking out of the endosome [47].

Eleven of the salt-responsive protein processing/trafficking components had increased expression due to the absence of *AtNHX1*, with seven of these transcripts not specific to a particular salt stress treatment, suggesting an influence of AtNHX1 over the entire range of the studied stress treatments.

The impact of *AtNHX1 *on vesicular trafficking is reflected by the altered expression of At1g22740, encoding RAB7, a small GTP-binding Ras-related protein, in the *nhx1 *line. Rab GTPases are part of the organization of intracellular membrane trafficking, including vesicle formation, vesicle motility, and vesicle tethering [48], and Rab7-related genes are important for the regulation of the late steps of endocytotic pathway. The overexpression of a *Rab7 *homolog stimulated endocytosis and conferred tolerance to salinity and oxidative stress in *Arabidopsis *[49,50]. Also a rice homologue of this gene was differentially regulated by both ABA and salinity and was implicated in vesicular traffic to the vacuole [51].

The altered expression pattern of an exocyst subunit EXO70 family protein (At5g59730) may be a further indication of the role of AtNHX1 in vesicular trafficking. Though not yet fully characterized in higher organisms, the EXO70 family members are important to vesicle docking and membrane fusion as well as regulation of actin polarity and transport of exocytic vesicles in yeast [52,53]. Also two kinesin-related transcripts (At5g47820 and At3g23670) showed an altered expression pattern. Kinesins are key to the intracellular transport system ([54] and references therein).

Four salt-responsive transcripts with roles in protein processing that are influenced by AtNHX1, emphasize the role of ion homeostasis on the proper folding and function of other proteins. These include two DnaJ-type genes (At2g20560 and At2g47440), a prefoldin (At1g08780), and a transducin/WD-40 repeat containing gene (At2g22040). The altered expression of these genes would suggest that the absence of AtNHX1 induces the instability of other proteins. Also, the altered expression of subtilases (At5g58810 and At4g34980) and a 26S proteasome regulatory subunit (RPN5/At5g64760) suggest a possible influence on protein degradation pathways.

A salt-responsive myosin XI subunit was also influenced by AtNHX1 (PCR43/XIC/At1g08730). Myosin XI mutants have been shown to be defective in both organelle movement and polar auxin transport [55] through the action on several vesicle-mediated processes. The altered expression of both a nuclear transport factor (NTF2/At3g25150) and a chloroplast outer membrane translocon subunit (At3g17970) would suggest a potential influence of AtNHX1 on trafficking of cellular components to organelles. Additionally, AtNHX1-influenced transcripts in other functional categories may also be related to a role of the antiporter as part of vesicular trafficking. For example, At2g17570, encoding a member of the undecaprenyl pyrophosphate synthetase family (Table 2C – Metabolism) is homologous to the yeast gene *RER2*, was shown to be important to vesicular processes and organelle integrity [56].

### Most salt-responsive transporters genes are not significantly influenced by *AtNHX1*

The Arabidopsis NHX family is comprised of 6 endomembrane (AtNHX1-6) and 2 plasma membrane-bound (AtNHX7/SOS1 and AtNHX8) members and in the absence of AtNHX1, compensation by the other AtNHX members might be expected, in particular when the plants are exposed to salt stress. However, our data did not show significant changes in the expression of any of the AtNHX2-8 transcripts either in *nhx1 *or NHX1::*nhx1 *plants in response to salt. Additionally, though the differences of AtNHX1 signal detection were at 27% and 160% of wild-type levels (p < 0.0001) for the *nhx1 *and NHX1::*nhx1 *lines, respectively, the other transporter genes did not show a significant difference of expression levels between lines regardless of the salt treatment used (data not shown).

A few salt-responsive transporters did show an apparent affect of AtNHX1 on expression levels (Table 2F). A putative phosphate transporter (At2g25520) showed an overall increased level of expression in the *nhx1 *plants, possibly as a result of an imbalance of phosphate ions as proton efflux from the vacuole is changed in the *nhx1 *line. A cyclic nucleotide-regulated ion channel (At2g23980/CNGC6) also showed increased expression in the *nhx1 *line. CNGCs comprise a family of 20 members in Arabidopsis, activated by direct binding of cyclic nucleotides and regulated by CaM [57]. They can provide a significant pathway for the non-selective uptake of ions (Na^+^, K^+ ^or Ca^2+^) and several family members were up-regulated or down-regulated by salt stress [58]. Since an increase in cellular cGMP was shown to occur during salt and osmotic stress [59], and the expression of *AtCNGC6 *was shown to be up-regulated in plants exposed to cGMP [60], it could be hypothesized that the overexpression of AtCNGC6 is related to the Na^+^-induced K^+^deficiency. Lastly, the expression of a nodulin-related gene (At1g31470) was increased in the *nhx1 *line with one week of salt stress, and the expression a cation efflux/metal tolerance family gene (At2g47830) was decreased with 48 hours of salt stress. The role of these putative transporters has yet to be elucidated.

Little is known about the influence of AtNHX1 on the expression/activity of other transporters within the plant cell. Previous work showed that *AtNHX1 *influenced the expression of a few genes encoding putative transporters [5]. However, as noted by Gong, et al. [61], previous microarray studies of salt stress in *Arabidopsis *(eg. [62,63]) did not demonstrate significantly altered expression of transporters, such as AtNHX1 or SOS1, which are known to contribute to ion homeostasis and salt tolerance [1,64]. Furthermore, a wide survey of available *Arabidopsis *microarray data suggested that only approximately 40 transcripts encoding putative cation transporters showed a significant response to salt or drought stress, with less than a 10% overlap between studies [58]. This emphasizes the influence of the experimental design on the expression profiles, suggesting a high level of inherent variability. Several factors might interfere with the detection of transcriptional changes in the genes encoding these transporters during salt stress, such as relatively low levels of expression or post-translational mechanisms that can modify the transporters affinity, selectivity, and/or its kinetics without affecting transcript expression [3,65].

## Conclusion

A unique feature of this study is the utilization of both an *nhx1 *'knockout' line and a 'rescued' mutant line (NHX1*::nhx1*) to identify transcripts with expression changes directly related to the presence of a single gene, *AtNHX1*. A previous study of the influence of *AtNHX1 *[5] on gene expression, was limited to only the *nhx1 *line in comparison to wild-type before and after the exposure of the plants to long-term (two weeks) salt stress. This work is a logical extension of the findings from the previous publication, because it provides novel aspects of the influence of the antiporter, especially as part of the salt stress response. We have provided evidence that *AtNHX1 *has a larger effect on salt responsive transcripts with increased salt stress duration rather than during the early exposure, emphasizing the increased importance of the antiporter during the later ionic effects of salt stress. Nonetheless the detection of AtNHX1-influenced salt-responsive transcripts during the earlier salt stress treatments, and the presence of 57 transcripts that appeared influenced regardless of any particular stress treatment, also highlights the role(s) of AtNHX1 throughout salt stress exposure. The use of short- and long-terms of sub-lethal levels of salt stress, together with the NHX1::*nhx1 *line, facilitated the elucidation of adaptive responses that are influenced by the vacuolar antiporter.

In line with its importance to salt stress tolerance, our results demonstrate that AtNHX1 influenced transcripts with known roles in the response to water deficit stress. We have additionally provided further evidence that AtNHX1 impacts the expression of other components of the response of Arabidopsis to stress. Recently, it has been shown that AtNHX1 activity can be modulated by calcium levels within the cell [3], and our results demonstrated that several Ca^2+^-binding elements were also affected transcriptionally by the presence of the antiporter protein. Furthermore, in addition to many uncharacterized transcripts, AtNHX1 also showed an impact on the transcription of several other key cellular processes including: sulfur metabolism, vesicular trafficking, protein processing, energy transfer processes, and cell growth/structure.

Up-regulation of most of the AtNHX1-influenced salt-responsive transcripts in the absence of AtNHX1 would suggest the activation of compensatory mechanisms in the *nhx1 *plants. Nevertheless, the decreased expression in transcripts encoding proteins with roles in metabolism and energy transfer would correlate with the phenotype displayed by the knockout plants, i.e. reduction of leaf area, smaller plants, and increased salt-sensitivity [4]. Also, the influence of AtNHX1 on vesicular trafficking and protein processing did not appear to be associated with any particular salt stress treatment, but rather appears to be an expression phenotype of the *nhx1 *plants, further indicating that, similar to its homolog in yeast [45-47], AtNHX1 plays an important role in ion and pH homeostasis of the cell endosomes.

The relatively small effect of *AtNHX1 *on the expression of other transporters during salt stress is noteworthy. Other microarray studies have also shown little impact of salt stress on the expression of ion transporters [62,63]. It could be argued that the non-lethal salt concentrations used here and in previous studies precluded the detection of significant changes in expression of transporters, and that under these conditions ion transport may be regulated primarily at the level of activity. Nevertheless, the *nhx1 *plants, in addition to being more sensitive to salt stress, are decreased in size, show developmental changes, and have decreased vacuolar H^+^-coupled cation transport [4]. This would indicate that any possible compensatory transport mechanism in the knockout plants was insufficient to maintain ion homeostasis at wild-type levels.

## Methods

### Plant materials and growth conditions

Three lines of *Arabidopsis thaliana *were used for this study, wild-type line (ecotype Wassilewskija; 'WS'), a 'knockout' line (*nhx1*) with a T-DNA insertion in the ninth exon of the *AtNHX1 *gene, and a 'rescued' line (*nhx1*::NHX1) with a single copy of the *AtNHX1 *coding sequence driven to constitutive expression by the 35S CMV promoter using the *nhx1 *line as the genetic background [4]. Seeds were surface sterilized with bleach and plated at an even density (~1 seed cm^-2^) in Petri dishes containing a modified MS growth medium supplemented with 8% agar and 5% sucrose. Seeds were germinated in an incubator (Model CU-36L; Percival Scientific, Perry, IA, USA) at 22°C under a 12-h photoperiod. Two weeks after sowing, seedlings of uniform size were selected and were transplanted into 100 ml pots (five seedlings per pot) containing moist soil mixture (MetroMix 200; Scotts Sierra Horticulture Products, Marysville, OH, USA). The pots were covered with a transparent plastic cover and placed in a growth chamber (Model AC-40 Controller 6000; Enconair, Winnipeg, MB, Canada) at 22°C under a short-day cycle (8 h light, 16 h dark) in order to delay bolting and enhance leaf development. Inflorescence tissues were removed nine days later (one week before harvest) to further emphasize leaf growth and to minimize developmental differences among plant lines and treatments. Plant were allowed to acclimatize for two days after transplanting and the soil was then saturated with the modified MS medium without or with supplemental 100 mM NaCl, as required. The watering solution was applied to the soil surface, allowed to drain and drainage was immediately removed to avoid salt accumulation.

Plants were subjected to salt stress for durations of 12-hours, 48-hours, 1-week or 2 weeks. The 2-week treatment was initiated after a 2-days acclimatization period, whereas other treatments were initiated afterwards at different times so that all treatments were harvested concurrently at the same age, 30 days after sowing. Plant material, excluding root and inflorescence tissues, was immediately frozen in liquid nitrogen for later expression analyses. Subsets of 25 plants (5 pots) of the same treatment and plant line were pooled to form an independent biological replicate. Four samples for control plants and three for each of the salt-stressed treatments were collected from each plant line.

### RNA extraction and GeneChip^® ^hybridization

Frozen plant samples were ground to a fine powder and RNA was extracted by a modification of the hot-phenol method [66]. After quality confirmation by agarose gel electrophoresis, the extracted RNA was prepared for array analysis as suggested by the manufacturer [67]. Briefly, ds-cDNA was made from total RNA, followed by formation of biotin-labeled cRNA, which was purified and fractionated prior to hybridization on individual gene chips. After overnight hybridization, the chips were stained with streptavidin-phycoerythrin and biotinylated anti-streptavidin antibody, then scanned by laser, producing an image file, the basis for quantifying and comparing relative transcript levels. Quantification of the data depends on a number of mathematical factors as optimized by Affymetrix [68] but is primarily based on the hybridization of experimental RNA to probe sets, each consisting of 11 representative 25-mer perfect match probes complementing unique portions of different transcripts and 11 corresponding single mismatch oligomer sequences. For this study, the Affymetrix^® ^ATH1-121501 Genome Array GeneChip^® ^was used, containing probe sets for 22,746 predicted and known expressed Arabidopsis genes.

### Data analysis

The data images produced by the microarray scanning were interpreted by Affymetrix^® ^Microarray Suite 5.0 (MAS 5.0) software with scaling of all probe sets to a target value of 500. The purpose of this chip-wide scaling was to minimize chip-to-chip difference in overall hybridization intensities [69]. A numerical file of all the data was produced and any transcript that did not generate a detection P-value <0.05 [70] for at least one chip was removed from the analysis (the default P-value cut-off for a 'present' expression call is 0.065). This filter eliminated 5,716 genes with unreliable expression data; because of low detection levels or non-specific probe sets. This also eliminated a large majority of transcripts with non-normal distribution of detection value data generated by MAS 5.0 algorithms [71]. Data from the remaining 17,030 genes were first normalized to an invariant set using dChip v1.2 computer software [72,73] and exported into Microsoft^® ^Excel^® ^(Microsoft Corp., Redmond, WA, USA) for further processing and analyses. Two statistical methods were used to identify salt responsive genes. A cross-wise log2 ratio analysis was performed with a cut-off threshold for significance set at two standard errors from a log change ratio of 0.585, corresponding to a 95% probability that the true mean represents at least a 50% deviation from the control treatment. In addition, one-tailed Student's homoscedastic t-tests with cutoff of P < 0.05 were used to evaluate the statistical significance of the difference between gene expression data under each salinity treatment vs. under the respective control. Twelve (3 plant lines × 4 salinity treatments) comparisons were made for each of the 17,030 genes. Only transcripts with comparisons that satisfied both statistical conditions under at least one salt treatment in at least one of the lines were retained for further analysis. These comparisons limited further analysis to the 4,027 transcripts that showed a significant response to salt treatment for at least one comparison. This approach to determine salt responsive transcripts, was used in a previous study of salt-treated Arabidopsis and verification by quantitative real-time PCR demonstrated the consistency of the method [5].

The selected salt-responsive genes were further analyzed to discover those that were influenced most strongly by the AtNHX1 antiporter. Of particular interest were gene transcripts either up- or down-regulated in the *nhx1 '*knockout' line, as compared to the wild-type line, with recovered expression levels in the NHX1::*nhx1 *'rescued' line. The 4,027 salt-responsive gene transcripts were subjected to a two-factor model analysis of variance using JMP software (SAS Institute, 2005). Gene transcripts showing a significant (p(F) < 0.05) line × treatment interaction (indicating treatment-dependent effect of plant line) or a significant main effect of the plant line (indicating difference between lines across all treatments) were subjected to means comparison by Student's t-test. Transcripts expression levels of the three plant lines were compared, either under each environment separately (for those showing significant interaction) or averaged across environments (for those showing a significant plant line effect but no significant interaction). A transcript was manifested as a salt-responsive AtNHX1-influenced gene if it exhibited a significantly decreased (down-regulated) or increased (up-regulated) level of expression in the *nhx1 *line relative to both the WS line and the NHX1*::nhx1 *line.

## Authors' contributions

JBS carried out the microarray studies, analyzed the data and drafted the manuscript; YS contributed to the data analysis and the preparation of the manuscript; EB contributed to the experimental design, data analysis and the final preparation of the manuscript. All authors have read and have approved the final manuscript.

## Supplementary Material

Additional file 1 Specific salt-responsive transcripts influenced by AtNHX1 that have an unclear cellular function Description : The 58 transcripts that met the same criteria as those found in Table 1 but that currently have an unclear functional classification Click here for file

## Figures and Tables

**Table 1:** Functional distribution of the 147 gene transcripts influenced by both salinity and AtNHX1.

**Gene classification**	**# of transcripts influenced under each treatment^1^**						**Distribution of decreased/increased transcripts in the *nhx1 *mutant^2^**	
		
	**All**	**Control**	**12 h**	**48 h**	**1wk**	**2wk**	**Down in *nhx1***	**Up in *nhx1***
Unclassified	25	5	7	5	9	9	12	46
DNA binding	4	4	3	4	3	3	5	16
Membrane Transport	1	1	0	1	1	0	1	3
Metabolism/Energy	12	0	3	6	2	2	16	9
Structure/Growth	2	1	1	3	4	2	4	9
Signaling	6	0	0	1	4	2	4	9
Protein Processing	8	1	1	1	1	2	3	11

**Total**	**58**	**12**	**15**	**21**	**24**	**20**	**45**	**103**

^1 ^three transcripts were specifically influenced by AtNHX1 under two treatments (At4g17120, At5g47490 – both unclassified, significantly affected by Control and 12 h treatments – and At3g54810 – DNA binding, significantly affected by 1wk and 2wk treatments)

^2 ^one transcript (At3g54810) was up-regulated in one treatment (1wk) and down regulated in a second treatment (2wk)

**Table 2:** Specific salt-responsive transcripts influenced by AtNHX1, organized by functional category

		**P(f)^a^**		**Treatment influenced by AtNHX1^b^**	**Transcripts intensity under the influenced treatment^c^**		
				
**Accession**	**Funtional Classes and Gene Descriptions**	**L**	**LxT**		*nhx1 *^d^	wild-type	NHX1::*nhx1*
	**A. DNA binding elements**						
At3g53730	histone H4		**	**Control**	2511.1	3461.3	4184.2
At5g67580	myb family transcription factor	*	*	**Control**	**287.4**	123.8	149.1
At5g35330	methyl-CpG-binding domain-containing protein	***	***	**Control**	**772.7**	526.9	508.3
At1g14685	BASIC PENTACYSTEINE 2, BPC2		**	**Control**	**555.1**	370.5	390.9
At2g36960	myb family transcription factor		*	**12 h**	298.0	368.5	476.0
At2g26150	heat shock transcription factor family protein		*	**12 h**	**684.1**	98.8	181.5
At3g24500	Transcriptional Coactivator Multiprotein Bridging Factor 1c.		*	**12 h**	**1024.0**	261.8	393.4
At1g69010	basic helix-loop-helix (bHLH) family protein	**	**	**48 h**	**422.3**	278.1	243.7
At3g56980	basic helix-loop-helix (bHLH) family protein		*	**48 h**	**503.2**	304.3	130.8
At4g25490	DRE-binding protein (DREB1B)/CRT/CRE-binding factor 1 (CBF1)		*	**48 h**	**758.3**	522.5	569.9
At1g69580	myb family transcription factor	**	*	**48 h**	**236.4**	141.3	135.7
At3g54810	zinc finger (GATA type) family protein^e^		**	**1 wk**	**1168.0**	571.7	340.6
At2g31730	putative ethylene-responsive protein		*	**1 wk**	**293.2**	147.8	47.1
At1g21910	DREB A-5 subfamily member, ERF/AP2 transcription factor family		*	**1 wk**	**1871.5**	771.5	598.6
At3g54810	zinc finger (GATA type) family protein^e^		**	**2 wk**	473.7	967.6	803.4
At4g00850	GRF1-interacting factor 3 (GIF3), SSXT family protein		**	**2 wk**	**366.7**	273.8	86.5
At2g04240	zinc finger (C3HC4-type RING finger) family protein		**	**2 wk**	**1018.2**	539.9	360.6
At5g57660	zinc finger (B-box type) family protein	*		**All**	1108.0	1585.0	1545.2
At4g27410	no apical meristem (NAM) family protein (RD26)	*		**All**	402.0	971.3	870.1
At5g56860	zinc finger (GATA type) family protein	***		**All**	**244.5**	157.7	125.6
At1g18710	myb family transcription factor (MYB47)	**		**All**	**257.3**	460.3	467.0

	**B. Signaling Elements**						
At4g34150	C2 domain-containing, similar to calcium-dependent protein kinase	***	**	**48 h**	2199.5	4215.4	4558.0
At4g08960	phosphotyrosyl phosphatase activator (PTPA) family protein	**	*	**1 wk**	**542.6**	377.1	279.9
At5g54380	protein kinase family protein		**	**1 wk**	**1866.1**	1250.7	826.4
At5g54840	GTP-binding family protein		**	**1 wk**	**134.1**	60.7	57.2
At5g66210	calcium-dependent protein kinase family protein (CPK28)		**	**1 wk**	**367.5**	221.8	213.5
At1g52570	phospholipase D alpha 2 (PLD2)/choline phosphatase 2		*	**2 wk**	**229.2**	99.4	74.5
At2g24160	pseudogene, leucine rich repeat protein family		*	**2 wk**	**349.8**	160.4	71.7
At2g38750	annexin 4 (ANN4)	***		**All**	511.2	875.1	804.2
At3g09960	calcineurin-like phosphoesterase family protein	*		**All**	59.2	98.7	104.2
At4g21370	putative S-locus protein kinase, pseudogene	*		**All**	63.0	102.8	110.1
At4g04540	protein kinase family protein///protein kinase family protein	**		**All**	**412.6**	289.1	220.3
At5g56040	leucine-rich repeat protein kinase, putative	**		**All**	**930.9**	748.2	617.4
At5g67380	casein kinase II alpha chain 1	***		**All**	**637.3**	496.1	488.6

	**C. Metabolism/Energy Components**						
At4g11600	putative glutathione peroxidase (AtGPX6)	**	*	**12 h**	3636.1	5433.9	4962.7
At1g68290	bifunctional nuclease, putative	***	*	**12 h**	105.1	236.2	244.6
At3g16050	putative pyridoxine (Vitamin B6) biosynthesis protein		*	**12 h**	**403.6**	121.1	227.3
At4g32360	NADP adrenodoxin-like ferredoxin reductase		*	**48 h**	102.5	172.0	203.7
At2g26560	putative patatin (PLP2)	***	**	**48 h**	1647.2	3298.0	3515.3
At1g56430	putative nicotianamine synthase		*	**48 h**	**995.2**	433.8	601.6
At3g03520	phosphoesterase family protein	**	*	**48 h**	**208.4**	125.2	122.2
At5g05960	protease inhibitor/seed storage/lipid transfer protein (LTP) family protein		*	**48 h**	**754.4**	423.3	436.3
At3g63440	FAD-binding domain-containing protein/cytokinin oxidase family protein		**	**48 h**	**224.6**	132.2	48.8
At4g04955	amidohydrolase family protein		*	**1 wk**	204.2	296.5	465.4
At1g63710	cytochrome P450, putative		**	**1 wk**	**126.9**	67.9	25.9
At2g17570	undecaprenyl pyrophosphate synthetase family protein		**	**2 wk**	112.5	206.2	310.0
At5g19890	putative peroxidase	*	**	**2 wk**	**212.8**	99.3	79.7
At1g28480	glutaredoxin family protein	***		**All**	349.1	696.8	1131.5
At2g46650	cytochrome b5, putative	**		**All**	1075.4	1748.2	1638.4
At5g17220	glutathione S-transferase (AtGSTF12, TT19)	*		**All**	270.8	402.7	422.1
At4g39940	adenylylsulfate kinase 2 (AKN2)	**		**All**	1940.2	2675.4	2748.4
At4g04610	5'-adenylylsulfate reductase (APR1)/PAPS reductase homolog (PRH19)	*		**All**	507.7	1362.0	1284.4
At3g22740	homocysteine S-methyltransferase 3 (HMT-3)	***		**All**	622.8	928.0	1155.4
At1g21440	mutase family protein	**		**All**	2176.8	2737.5	2793.7
At1g06520	phospholipid/glycerol acyltransferase family protein	**		**All**	69.9	120.1	126.8
At1g16410	cytochrome P450 family protein (CYP79F1) (bushy1)	***		**All**	280.2	480.4	492.0
At2g32150	haloacid dehalogenase-like hydrolase family protein	***		**All**	357.9	720.0	857.9
At5g47240	MutT/nudix family protein	***		**All**	961.5	1939.5	1471.4
At2g06050	12-oxophytodienoate reductase (OPR3)/delayed dehiscence1 (DDE1)	**		**All**	804.0	1315.1	1454.0

	**D. Structure/Growth Components**						
At2g20000	cell division cycle family protein/CDC family protein		*	**Control**	**429.7**	274.3	260.2
At2g40610	expansin, putative (EXP8)	*	*	**12 hours**	**792.4**	462.8	381.2
At1g27630	cyclin family protein		**	**48 h**	519.1	744.7	913.9
At3g02350	glycosyl transferase family 8 protein	*	*	**48 h**	**1160.2**	947.2	609.2
At1g19170	glycoside hydrolase family 28/polygalacturonase (pectinase) family	*	*	**48 h**	**365.0**	220.3	177.9
At3g45970	expansin family protein (EXPL1/AtEXLA1)		*	**1 wk**	**3701.0**	1684.2	1026.0
At3g62720	galactosyl transferase GMA12/MNN10 family protein		**	**1 wk**	**2237.1**	1459.9	790.3
At5g57560	cell wall-modifying enzyme, endo-xyloglucan transferase (TCH4)		**	**1 wk**	**13493.5**	6314.8	6047.0
At4g30470	cinnamoyl-CoA reductase-related		*	**1 wk**	**756.6**	478.9	512.6
At1g57590	putative pectinacetylesterase	*	*	**2 wk**	143.8	381.3	336.7
At1g16340	putative 3-deoxy-D-manno-2-octulosonate-8-phosphate synthase		*	**2 wk**	**352.0**	231.3	48.7
At4g16590	glucosyltransferase-related	*		**All**	194.5	578.7	602.4
At1g24070	glycosyl transferase family 2 protein (AtCSLA10)	**		**All**	280.2	509.7	495.6

	**E. Protein Processing**						
At3g17970	chloroplast outer membrane translocon subunit, putative		*	**Control**	**252.5**	115.4	162.0
At2g20560	DNAJ heat shock family protein		*	**12 h**	**413.3**	159.6	155.0
At1g08780	prefoldin, putative		*	**48 h**	**476.4**	303.2	182.0
At2g47440	DNAJ heat shock N-terminal domain-containing protein		**	**1 wk**	**3769.5**	2247.3	1271.8
At1g08730	myosin heavy chain (PCR43) (XIC)	***	***	**2 wk**	25.4	147.4	204.6
At5g58810	subtilisin-like serine protease, similar to prepro-cucumisin	***	**	**2 wk**	24.0	148.8	201.3
At5g59730	exocyst subunit EXO70 family protein	*		**All**	768.8	1096.8	1199.6
At3g25150	nuclear transport factor 2 (NTF2) family protein	***		**All**	**693.3**	527.6	439.1
At5g64760	26S proteasome regulatory subunit, putative (RPN5)	**		**All**	**419.3**	340.3	316.2
At1g22740	Ras-related protein (RAB7)/AtRab75/small GTP-binding	***		**All**	**1324.9**	892.1	703.5
At2g22040	transducin family protein/WD-40 repeat family protein	***		**All**	**362.1**	283.2	231.5
At5g47820	kinesin-like protein (FRA1)	**		**All**	**389.3**	303.8	259.8
At4g34980	subtilase family protein (SLP2)	**		**All**	**1133.4**	899.5	930.4
At3g23670	phragmoplast-associated kinesin-related protein, putative	**		**All**	**138.2**	101.9	75.6

	**F. Membrane Transport**						
At2g23980	cyclic nucleotide-regulated ion channel (CNGC6)	*	*	**Control**	**393.3**	259.6	180.7
At2g47830	cation efflux family/metal tolerance (MTPc1)		**	**48 h**	73.9	149.7	166.0
At1g31470	nodulin-related		*	**1 wk**	**223.1**	148.5	110.3
At2g25520	phosphate translocator-related	**		**All**	**999.7**	790.3	773.9

^a ^*, ** and *** indicate significant F values for the plant line effect and line × treatment interaction at the 0.05, 0.01 and 0.001 levels, respectively. An additional 58 salt-responsive AtNHX1 influenced transcripts with unclear functional assignment are not presented and can be found in Additional File 1

^b ^the specific treatment influenced by AtNHX1 for cases of significant interaction, or 'All' for cases where only the plant line effect was significant.

^c ^transcript intensity of the three plant lines for the treatment of interest, with the average expression value of all treatments used when only plant line effect was significant.

^d ^transcript intensity of the *nhx1 *line is in bold font for cases where the expression level is higher compared to the other lines, normal font signifies reduced expression.

^e ^At3g54810 is represented twice because it showed a significant influence of AtNHX1 at both one week and two weeks of salt treatment, with alternate relative levels of expression of the *nhx1 *line
