# Supplementary material for: Impact of AtNHX1, a vacuolar Na+/H+ antiporter, upon gene expression during short- and long-term salt stress in Arabidopsis thaliana
Source: BMC Plant Biol. 2007 Apr 5;7:18. doi: 10.1186/1471-2229-7-18 (PMC1853094; doi:10.1186/1471-2229-7-18)
Supplement: Additional file 1 — Specific salt-responsive transcripts influenced by AtNHX1 that have an unclear cellular function Description : The 58 transcripts that met the same criteria as those found in Table 1 but that currently have an unclear functional classification [file 1471-2229-7-18-S1.pdf]

Additional File 1.
Specific salt-responsive transcripts influenced by AtNHX1 that have an unclear cellular function

		P(f)[a]		Treatment influenced by	Transcripts intensity under the influenced treatment		
**Accession**	**Gene Descriptions**	**L**	**LxT**	**AtNHX1[b]**	*nhx1* [c]	wild-type	NHX1::*nhx1*
At2g37240	expressed protein	***	**	**Control**	239.5	419.8	418.4
At5g01400	expressed protein (similar to symplekin)	*	**	**Control**	266.2	381.7	421.2
At1g10020	expressed protein	**	**	**Control**	**849.4**	508.3	394.5
At4g17120	expressed protein[d]	**	*	**Control**	**536.6**	270.3	297.6
At3g02420	expressed protein		*	**Control**	**960.6**	720.2	729.8
At5g66930	expressed protein	**	*	**12h**	**452.8**	289.4	311.3
At1g66840	expressed protein	*	*	**12h**	**134.6**	77.0	68.6
At5g47490	expressed protein[d]		**	**12h**	**169.1**	114.6	37.5
At4g17120	expressed protein[d]	**	*	**12h**	**418.6**	183.2	194.4
At2g32690	pseudogene		*	**12h**	**330.9**	92.6	121.0
At2g36480	zinc finger (C2H2-type) family protein		*	**12h**	**231.7**	119.0	103.2
At5g07290	RNA recognition motif (RRM)-containing	*	*	**12h**	**442.2**	225.3	213.5
At1g61830	pseudogene, putative CHP-rich zinc finger protein	***	**	**48h**	38.1	61.4	93.8
At1g20070	expressed protein	*	***	**48h**	**363.6**	131.5	164.6
At1g31460	expressed protein		*	**48h**	**328.4**	155.6	100.1
At3g32270	hypothetical protein		*	**48h**	**198.1**	70.2	41.1
At3g54260	expressed protein		*	**48h**	**978.3**	554.3	646.3
At5g58510	expressed protein	**	**	**1 wk**	**327.3**	170.2	78.3
At1g68440	expressed protein	**	*	**1 wk**	**443.5**	263.8	70.0
At3g58620	tetratricopeptide repeat (TPR)-containing protein	*	*	**1 wk**	**448.3**	326.1	98.3
At4g08950	putative phosphate-responsive (EXO)		*	**1 wk**	**8943.5**	4193.8	2951.2
At1g35140	putative phosphate-responsive protein		*	**1 wk**	**1914.6**	604.1	372.0
At2g17230	phosphate-responsive 1 family protein		*	**1 wk**	**2779.3**	1931.6	1293.8
At3g59310	anthocyanin-related membrane protein 1 (Anm1)	***	**	**1 wk**	**1516.8**	1048.1	313.7
At2g47770	benzodiazepine receptor-related		**	**1 wk**	**292.2**	86.1	128.7
At5g66200	armadillo/beta-catenin repeat family		**	**1 wk**	**1324.2**	981.9	773.5
At5g47490	expressed protein[d]		**	**2 wk**	**304.4**	191.4	162.2
At3g62300	agenet domain-containing		*	**2 wk**	**241.7**	116.6	49.0
At4g08730	hypothetical protein	*	*	**2 wk**	**189.2**	121.1	84.4
At4g35130	pentatricopeptide (PPR) repeat-containing		*	**2 wk**	**166.3**	56.2	72.4
At3g12950	expressed protein	***	*	**2 wk**	**299.0**	142.7	63.3
At5g60590	yrdC protein-related		***	**2 wk**	**258.3**	93.8	69.8
At4g14220	zinc finger (C3HC4-type RING finger) family	**	**	**2 wk**	**345.0**	188.8	92.2
At5g66260	putative auxin-responsive	*	**	**2 wk**	**194.1**	84.5	85.0
At3g45830	expressed protein	*	**	**2 wk**	**388.6**	115.1	240.4
At1g72450	expressed protein	***		**All**	1230.0	1714.7	2356.8
At5g55640	expressed protein	***		**All**	291.5	413.8	407.5
At5g13220	expressed protein	*		**All**	263.1	468.6	521.2
At4g33666	expressed protein	*		**All**	1592.6	2020.5	2066.1
At1g49500	expressed protein	*		**All**	4340.3	6299.8	6294.3
At1g03055	expressed protein	**		**All**	101.3	158.0	184.3
At1g03820	expressed protein	***		**All**	443.4	556.3	588.7
At1g23180	armadillo/beta-catenin repeat family	*		**All**	441.0	557.9	543.0
At3g01920	yrdC family protein	*		**All**	334.1	448.7	442.3
At1g53885	senescence-associated protein-related	**		**All**	111.1	284.9	267.6
At5g12050	expressed protein	**		**All**	**265.6**	143.1	195.6
At4g34830	similar to salt-inducible protein from tobacco, PPR repeat-containing	**		**All**	**343.9**	207.2	169.7
At3g19540	expressed protein	**		**All**	**606.3**	452.4	459.5
At1g21580	hydroxyproline-rich glycoprotein family protein	*		**All**	**186.2**	135.2	126.6
At2g28310	expressed protein	**		**All**	**318.3**	240.9	192.2
At2g34510	expressed protein	***		**All**	**2502.7**	1739.7	1478.3
At1g11780	oxidoreductase, 2OG-Fe(II) oxygenase family protein	*		**All**	**275.0**	219.2	204.6
At1g78930	mitochondrial transcription termination factor-related / mTERF-related	*		**All**	**232.5**	159.6	166.8
At5g07950	expressed protein	*		**All**	**139.6**	78.5	84.1
At1g18580	glycosyltransferase family protein 8	*		**All**	**345.8**	248.2	249.1
At5g45510	leucine-rich repeat family protein	*		**All**	**776.4**	574.0	521.2
At5g52890	AT hook motif-containing protein	*		**All**	**86.5**	57.6	46.9
At4g12640	RNA recognition motif (RRM)-containing protein	**		**All**	**223.1**	147.4	146.2
At1g33470	RNA recognition motif (RRM)-containing protein	*		**All**	**167.9**	87.3	114.2
At5g08500	transmembrane CLPTM1 family protein	***		**All**	**665.0**	487.5	454.8

[a] *, ** and *** indicate significant F values for the plant line effect and line x treatment interaction at the 0.05, 0.01 and 0.001 levels, respectively

[b] the specific treatment influenced by AtNHX1 for cases of significant interaction, or 'All' for cases where only the plant line effect was significant.

[c] transcript intensity of the *nhx1* line is in bold font for cases where the expression level is higher compared to the other lines, normal font signifies reduced expression.

[d] two transcripts (At4g17120 and At5g47490) showed a signficant influence of *AtNHX1* with two different durations of salt stress.
